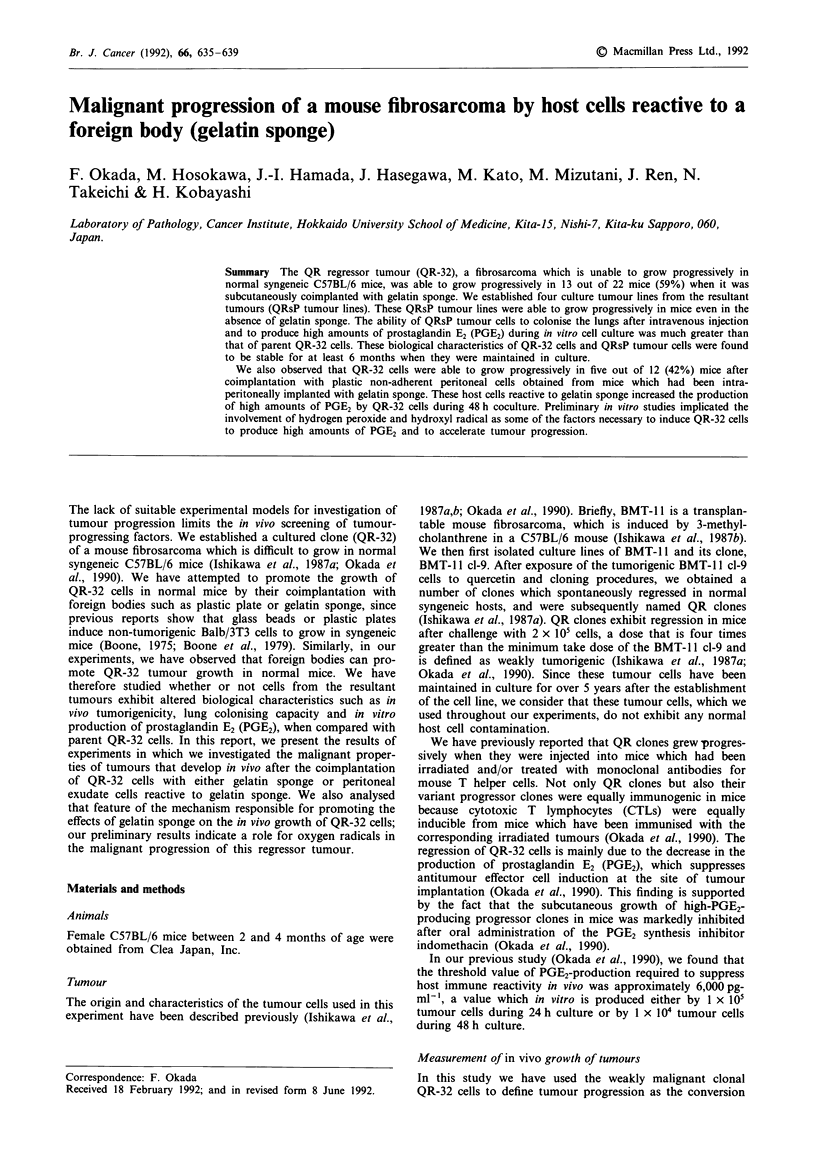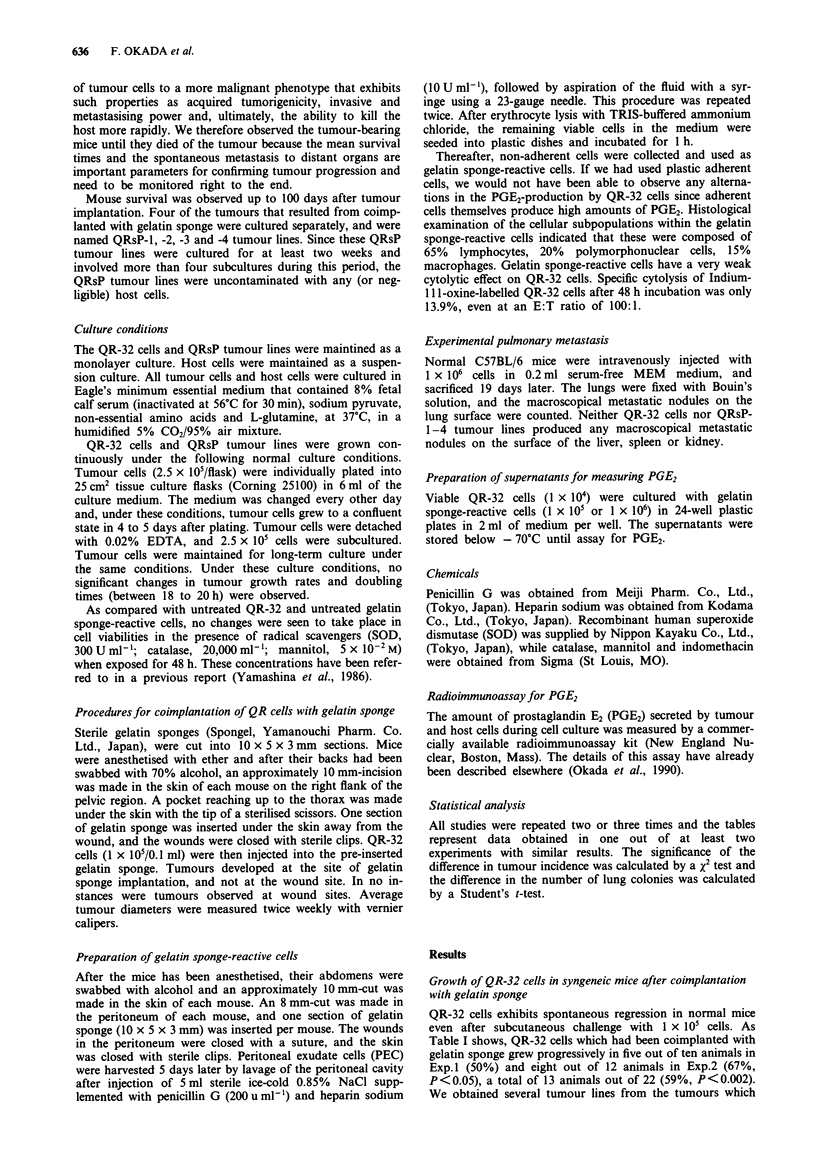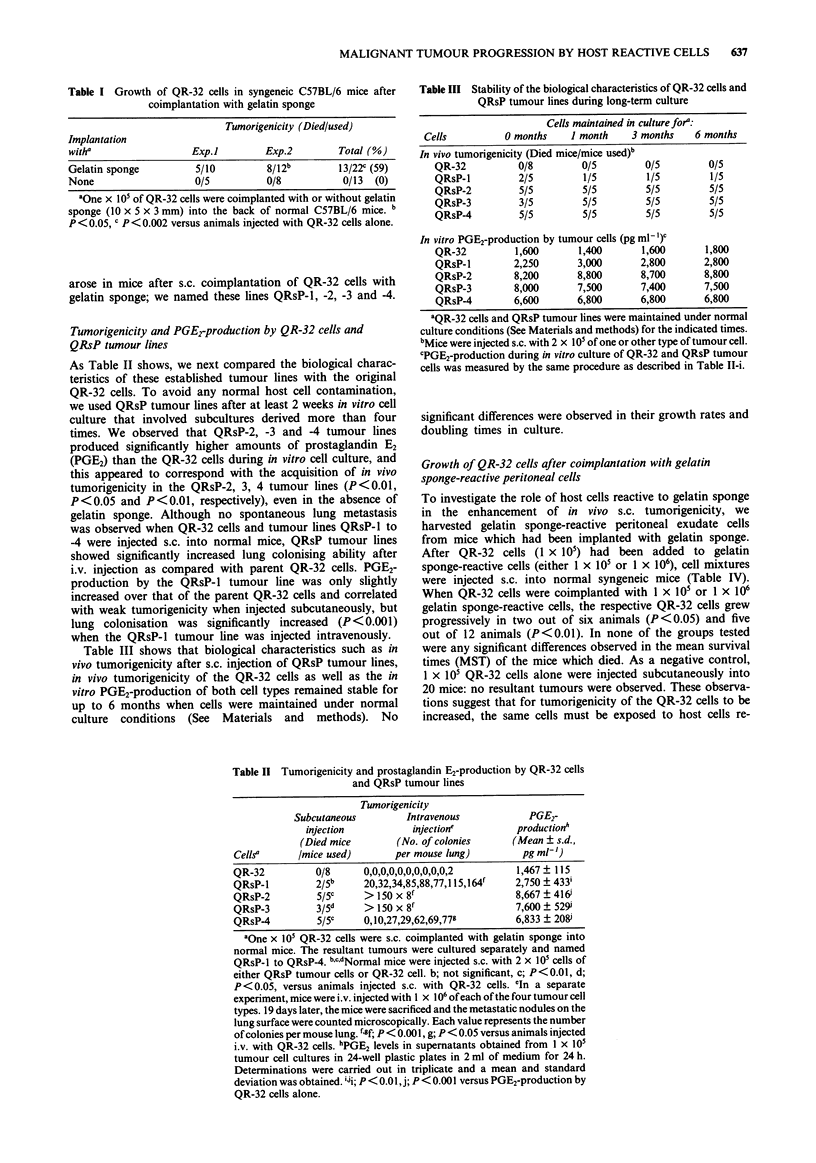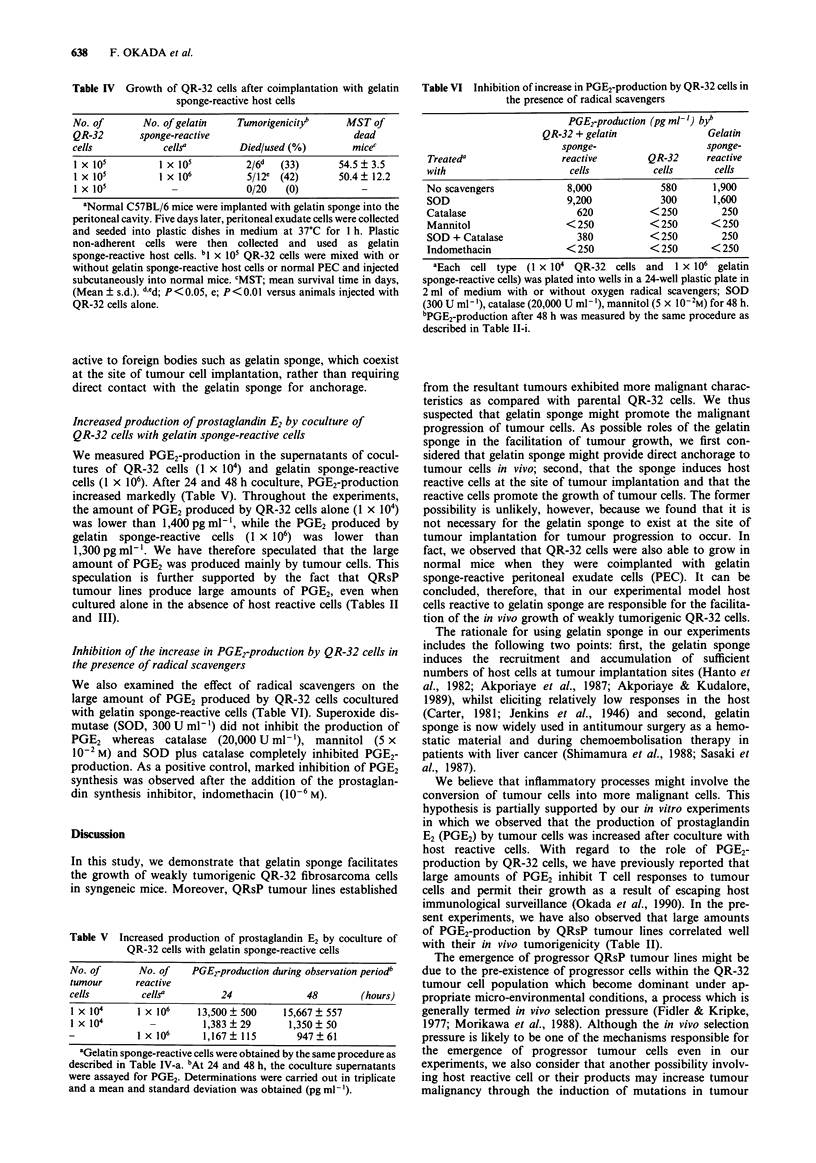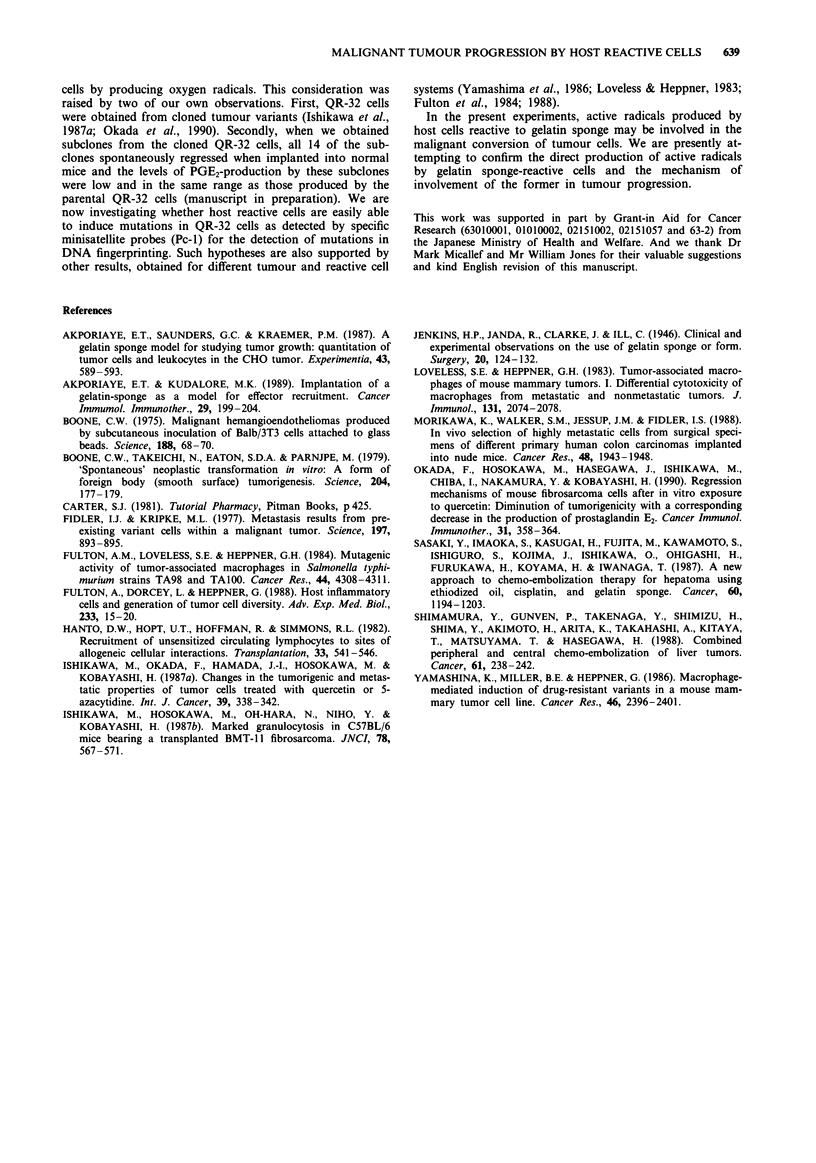# Malignant progression of a mouse fibrosarcoma by host cells reactive to a foreign body (gelatin sponge).

**DOI:** 10.1038/bjc.1992.329

**Published:** 1992-10

**Authors:** F. Okada, M. Hosokawa, J. I. Hamada, J. Hasegawa, M. Kato, M. Mizutani, J. Ren, N. Takeichi, H. Kobayashi

**Affiliations:** Laboratory of Pathology, Hokkaido University School of Medicine, Sapporo, Japan.

## Abstract

The QR regressor tumour (QR-32), a fibrosarcoma which is unable to grow progressively in normal syngeneic C57BL/6 mice, was able to grow progressively in 13 out of 22 mice (59%) when it was subcutaneously coimplanted with gelatin sponge. We established four culture tumour lines from the resultant tumours (QRsP tumour lines). These QRsP tumour lines were able to grow progressively in mice even in the absence of gelatin sponge. The ability of QRsP tumour cells to colonise the lungs after intravenous injection and to produce high amounts of prostaglandin E2 (PGE2) during in vitro cell culture was much greater than that of parent QR-32 cells. These biological characteristics of QR-32 cells and QRsP tumour cells were found to be stable for at least 6 months when they were maintained in culture. We also observed that QR-32 cells were able to grow progressively in five out of 12 (42%) mice after coimplantation with plastic non-adherent peritoneal cells obtained from mice which had been intraperitoneally implanted with gelatin sponge. These host cells reactive to gelatin sponge increased the production of high amounts of PGE2 by QR-32 cells during 48 h coculture. Preliminary in vitro studies implicated the involvement of hydrogen peroxide and hydroxyl radical as some of the factors necessary to induce QR-32 cells to produce high amounts of PGE2 and to accelerate tumour progression.


Br. J. Cancer (1992), 66, 635–639 © Macmillan Press Ltd., 1992

### Malignant progression of a mouse fibrosarcoma by host cells reactive to a foreign body (gelatin sponge)

F. Okada, M. Hosokawa, J.-I. Hamada, J. Hasegawa, M. Kato, M. Mizutani, J. Ren, N. Takeichi & H. Kobayashi

*Laboratory of Pathology, Cancer Institute, Hokkaido University School of Medicine, Kita-15, Nishi-7, Kita-ku Sapporo, 060, Japan.*

**Summary** The QR regressor tumour (QR-32), a fibrosarcoma which is unable to grow progressively in normal syngeneic C57BL/6 mice, was able to grow progressively in 13 out of 22 mice (59%) when it was subcutaneously coimplanted with gelatin sponge. We established four culture tumour lines from the resultant tumours (QRsP tumour lines). These QRsP tumour lines were able to grow progressively in mice even in the absence of gelatin sponge. The ability of QRsP tumour cells to colonise the lungs after intravenous injection and to produce high amounts of prostaglandin $E_2$ ($PGE_2$) during *in vitro* cell culture was much greater than that of parent QR-32 cells. These biological characteristics of QR-32 cells and QRsP tumour cells were found to be stable for at least 6 months when they were maintained in culture.

We also observed that QR-32 cells were able to grow progressively in five out of 12 (42%) mice after coimplantation with plastic non-adherent peritoneal cells obtained from mice which had been intraperitoneally implanted with gelatin sponge. These host cells reactive to gelatin sponge increased the production of high amounts of $PGE_2$ by QR-32 cells during 48 h coculture. Preliminary *in vitro* studies implicated the involvement of hydrogen peroxide and hydroxyl radical as some of the factors necessary to induce QR-32 cells to produce high amounts of $PGE_2$ and to accelerate tumour progression.

The lack of suitable experimental models for investigation of tumour progression limits the *in vivo* screening of tumour-progressing factors. We established a cultured clone (QR-32) of a mouse fibrosarcoma which is difficult to grow in normal syngeneic C57BL/6 mice (Ishikawa *et al.*, 1987*a*; Okada *et al.*, 1990). We have attempted to promote the growth of QR-32 cells in normal mice by their coimplantation with foreign bodies such as plastic plate or gelatin sponge, since previous reports show that glass beads or plastic plates induce non-tumorigenic Balb/3T3 cells to grow in syngeneic mice (Boone, 1975; Boone *et al.*, 1979). Similarly, in our experiments, we have observed that foreign bodies can promote QR-32 tumour growth in normal mice. We have therefore studied whether or not cells from the resultant tumours exhibit altered biological characteristics such as *in vivo* tumorigenicity, lung colonising capacity and *in vitro* production of prostaglandin $E_2$ ($PGE_2$), when compared with parent QR-32 cells. In this report, we present the results of experiments in which we investigated the malignant properties of tumours that develop *in vivo* after the coimplantation of QR-32 cells with either gelatin sponge or peritoneal exudate cells reactive to gelatin sponge. We also analysed that feature of the mechanism responsible for promoting the effects of gelatin sponge on the *in vivo* growth of QR-32 cells; our preliminary results indicate a role for oxygen radicals in the malignant progression of this regressor tumour.

### Materials and methods

### *Animals*

Female C57BL/6 mice between 2 and 4 months of age were obtained from Clea Japan, Inc.

### *Tumour*

The origin and characteristics of the tumour cells used in this experiment have been described previously (Ishikawa *et al.*, 1987*a*,*b*; Okada *et al.*, 1990). Briefly, BMT-11 is a transplantable mouse fibrosarcoma, which is induced by 3-methylcholanthrene in a C57BL/6 mouse (Ishikawa *et al.*, 1987*b*). We then first isolated culture lines of BMT-11 and its clone, BMT-11 cl-9. After exposure of the tumorigenic BMT-11 cl-9 cells to quercetin and cloning procedures, we obtained a number of clones which spontaneously regressed in normal syngeneic hosts, and were subsequently named QR clones (Ishikawa *et al.*, 1987*a*). QR clones exhibit regression in mice after challenge with $2 \times 10^5$ cells, a dose that is four times greater than the minimum take dose of the BMT-11 cl-9 and is defined as weakly tumorigenic (Ishikawa *et al.*, 1987*a*; Okada *et al.*, 1990). Since these tumour cells have been maintained in culture for over 5 years after the establishment of the cell line, we consider that these tumour cells, which we used throughout our experiments, do not exhibit any normal host cell contamination.

We have previously reported that QR clones grew progressively when they were injected into mice which had been irradiated and/or treated with monoclonal antibodies for mouse T helper cells. Not only QR clones but also their variant progressor clones were equally immunogenic in mice because cytotoxic T lymphocytes (CTLs) were equally inducible from mice which have been immunised with the corresponding irradiated tumours (Okada *et al.*, 1990). The regression of QR-32 cells is mainly due to the decrease in the production of prostaglandin $E_2$ ($PGE_2$), which suppresses antitumour effector cell induction at the site of tumour implantation (Okada *et al.*, 1990). This finding is supported by the fact that the subcutaneous growth of high-$PGE_2$-producing progressor clones in mice was markedly inhibited after oral administration of the $PGE_2$ synthesis inhibitor indomethacin (Okada *et al.*, 1990).

In our previous study (Okada *et al.*, 1990), we found that the threshold value of $PGE_2$-production required to suppress host immune reactivity *in vivo* was approximately 6,000 pg-$ml^{-1}$, a value which *in vitro* is produced either by $1 \times 10^5$ tumour cells during 24 h culture or by $1 \times 10^4$ tumour cells during 48 h culture.

### *Measurement of* in vivo *growth of tumours*

In this study we have used the weakly malignant clonal QR-32 cells to define tumour progression as the conversion

Correspondence: F. Okada
Received 18 February 1992; and in revised form 8 June 1992.

of tumour cells to a more malignant phenotype that exhibits such properties as acquired tumorigenicity, invasive and metastasising power and, ultimately, the ability to kill the host more rapidly. We therefore observed the tumour-bearing mice until they died of the tumour because the mean survival times and the spontaneous metastasis to distant organs are important parameters for confirming tumour progression and need to be monitored right to the end.

Mouse survival was observed up to 100 days after tumour implantation. Four of the tumours that resulted from coimplanted with gelatin sponge were cultured separately, and were named QRsP-1, -2, -3 and -4 tumour lines. Since these QRsP tumour lines were cultured for at least two weeks and involved more than four subcultures during this period, the QRsP tumour lines were uncontaminated with any (or negligible) host cells.

*Culture conditions*

The QR-32 cells and QRsP tumour lines were maintined as a monolayer culture. Host cells were maintained as a suspension culture. All tumour cells and host cells were cultured in Eagle's minimum essential medium that contained 8% fetal calf serum (inactivated at 56°C for 30 min), sodium pyruvate, non-essential amino acids and L-glutamine, at 37°C, in a humidified 5% $CO_2$/95% air mixture.

QR-32 cells and QRsP tumour lines were grown continuously under the following normal culture conditions. Tumour cells ($2.5 \times 10^5$/flask) were individually plated into 25 $cm^2$ tissue culture flasks (Corning 25100) in 6 ml of the culture medium. The medium was changed every other day and, under these conditions, tumour cells grew to a confluent state in 4 to 5 days after plating. Tumour cells were detached with 0.02% EDTA, and $2.5 \times 10^5$ cells were subcultured. Tumour cells were maintained for long-term culture under the same conditions. Under these culture conditions, no significant changes in tumour growth rates and doubling times (between 18 to 20 h) were observed.

As compared with untreated QR-32 and untreated gelatin sponge-reactive cells, no changes were seen to take place in cell viabilities in the presence of radical scavengers (SOD, 300 U $ml^{-1}$; catalase, 20,000 $ml^{-1}$; mannitol, $5 \times 10^{-2}$ M) when exposed for 48 h. These concentrations have been referred to in a previous report (Yamashina *et al.*, 1986).

*Procedures for coimplantation of QR cells with gelatin sponge*

Sterile gelatin sponges (Spongel, Yamanouchi Pharm. Co. Ltd., Japan), were cut into 10 × 5 × 3 mm sections. Mice were anesthetised with ether and after their backs had been swabbed with 70% alcohol, an approximately 10 mm-incision was made in the skin of each mouse on the right flank of the pelvic region. A pocket reaching up to the thorax was made under the skin with the tip of a sterilised scissors. One section of gelatin sponge was inserted under the skin away from the wound, and the wounds were closed with sterile clips. QR-32 cells ($1 \times 10^5$/0.1 ml) were then injected into the pre-inserted gelatin sponge. Tumours developed at the site of gelatin sponge implantation, and not at the wound site. In no instances were tumours observed at wound sites. Average tumour diameters were measured twice weekly with vernier calipers.

*Preparation of gelatin sponge-reactive cells*

After the mice has been anesthetised, their abdomens were swabbed with alcohol and an approximately 10 mm-cut was made in the skin of each mouse. An 8 mm-cut was made in the peritoneum of each mouse, and one section of gelatin sponge (10 × 5 × 3 mm) was inserted per mouse. The wounds in the peritoneum were closed with a suture, and the skin was closed with sterile clips. Peritoneal exudate cells (PEC) were harvested 5 days later by lavage of the peritoneal cavity after injection of 5 ml sterile ice-cold 0.85% NaCl supplemented with penicillin G (200 u $ml^{-1}$) and heparin sodium (10 U $ml^{-1}$), followed by aspiration of the fluid with a syringe using a 23-gauge needle. This procedure was repeated twice. After erythrocyte lysis with TRIS-buffered ammonium chloride, the remaining viable cells in the medium were seeded into plastic dishes and incubated for 1 h.

Thereafter, non-adherent cells were collected and used as gelatin sponge-reactive cells. If we had used plastic adherent cells, we would not have been able to observe any alternations in the $PGE_2$-production by QR-32 cells since adherent cells themselves produce high amounts of $PGE_2$. Histological examination of the cellular subpopulations within the gelatin sponge-reactive cells indicated that these were composed of 65% lymphocytes, 20% polymorphonuclear cells, 15% macrophages. Gelatin sponge-reactive cells have a very weak cytolytic effect on QR-32 cells. Specific cytolysis of Indium-111-oxine-labelled QR-32 cells after 48 h incubation was only 13.9%, even at an E:T ratio of 100:1.

*Experimental pulmonary metastasis*

Normal C57BL/6 mice were intravenously injected with $1 \times 10^6$ cells in 0.2 ml serum-free MEM medium, and sacrificed 19 days later. The lungs were fixed with Bouin's solution, and the macroscopical metastatic nodules on the lung surface were counted. Neither QR-32 cells nor QRsP-1–4 tumour lines produced any macroscopical metastatic nodules on the surface of the liver, spleen or kidney.

*Preparation of supernatants for measuring $PGE_2$*

Viable QR-32 cells ($1 \times 10^4$) were cultured with gelatin sponge-reactive cells ($1 \times 10^5$ or $1 \times 10^6$) in 24-well plastic plates in 2 ml of medium per well. The supernatants were stored below − 70°C until assay for $PGE_2$.

*Chemicals*

Penicillin G was obtained from Meiji Pharm. Co., Ltd., (Tokyo, Japan). Heparin sodium was obtained from Kodama Co., Ltd., (Tokyo, Japan). Recombinant human superoxide dismutase (SOD) was supplied by Nippon Kayaku Co., Ltd., (Tokyo, Japan), while catalase, mannitol and indomethacin were obtained from Sigma (St Louis, MO).

*Radioimmunoassay for $PGE_2$*

The amount of prostaglandin $E_2$ ($PGE_2$) secreted by tumour and host cells during cell culture was measured by a commercially available radioimmunoassay kit (New England Nuclear, Boston, Mass). The details of this assay have already been described elsewhere (Okada *et al.*, 1990).

*Statistical analysis*

All studies were repeated two or three times and the tables represent data obtained in one out of at least two experiments with similar results. The significance of the difference in tumour incidence was calculated by a $\chi^2$ test and the difference in the number of lung colonies was calculated by a Student's *t*-test.

### Results

*Growth of QR-32 cells in syngeneic mice after coimplantation with gelatin sponge*

QR-32 cells exhibits spontaneous regression in normal mice even after subcutaneous challenge with $1 \times 10^5$ cells. As Table I shows, QR-32 cells which had been coimplanted with gelatin sponge grew progressively in five out of ten animals in Exp.1 (50%) and eight out of 12 animals in Exp.2 (67%, $P < 0.05$), a total of 13 animals out of 22 (59%, $P < 0.002$). We obtained several tumour lines from the tumours which

**Table I** Growth of QR-32 cells in syngeneic C57BL/6 mice after coimplantation with gelatin sponge

| Implantation with[a] | Tumorigenicity (Died/used) Exp.1 | Exp.2 | Total (%) |
|---|---|---|---|
| Gelatin sponge | 5/10 | 8/12[b] | 13/22[c] (59) |
| None | 0/5 | 0/8 | 0/13 (0) |

[a]One × $10^5$ of QR-32 cells were coimplanted with or without gelatin sponge (10 × 5 × 3 mm) into the back of normal C57BL/6 mice. [b] $P<0.05$, [c] $P<0.002$ versus animals injected with QR-32 cells alone.

arose in mice after s.c. coimplantation of QR-32 cells with gelatin sponge; we named these lines QRsP-1, -2, -3 and -4.

*Tumorigenicity and $PGE_2$-production by QR-32 cells and QRsP tumour lines*

As Table II shows, we next compared the biological characteristics of these established tumour lines with the original QR-32 cells. To avoid any normal host cell contamination, we used QRsP tumour lines after at least 2 weeks *in vitro* cell culture that involved subcultures derived more than four times. We observed that QRsP-2, -3 and -4 tumour lines produced significantly higher amounts of prostaglandin $E_2$ ($PGE_2$) than the QR-32 cells during *in vitro* cell culture, and this appeared to correspond with the acquisition of *in vivo* tumorigenicity in the QRsP-2, 3, 4 tumour lines ($P<0.01$, $P<0.05$ and $P<0.01$, respectively), even in the absence of gelatin sponge. Although no spontaneous lung metastasis was observed when QR-32 cells and tumour lines QRsP-1 to -4 were injected s.c. into normal mice, QRsP tumour lines showed significantly increased lung colonising ability after i.v. injection as compared with parent QR-32 cells. $PGE_2$-production by the QRsP-1 tumour line was only slightly increased over that of the parent QR-32 cells and correlated with weak tumorigenicity when injected subcutaneously, but lung colonisation was significantly increased ($P<0.001$) when the QRsP-1 tumour line was injected intravenously.

Table III shows that biological characteristics such as *in vivo* tumorigenicity after s.c. injection of QRsP tumour lines, *in vivo* tumorigenicity of the QR-32 cells as well as the *in vitro* $PGE_2$-production of both cell types remained stable for up to 6 months when cells were maintained under normal culture conditions (See Materials and methods). No

**Table III** Stability of the biological characteristics of QR-32 cells and QRsP tumour lines during long-term culture

| Cells | Cells maintained in culture for[a]: 0 months | 1 month | 3 months | 6 months |
|---|---|---|---|---|
| *In vivo* tumorigenicity (Died mice/mice used)[b] | | | | |
| QR-32 | 0/8 | 0/5 | 0/5 | 0/5 |
| QRsP-1 | 2/5 | 1/5 | 1/5 | 1/5 |
| QRsP-2 | 5/5 | 5/5 | 5/5 | 5/5 |
| QRsP-3 | 3/5 | 5/5 | 5/5 | 5/5 |
| QRsP-4 | 5/5 | 5/5 | 5/5 | 5/5 |
| *In vitro* $PGE_2$-production by tumour cells (pg $ml^{-1}$)[c] | | | | |
| QR-32 | 1,600 | 1,400 | 1,600 | 1,800 |
| QRsP-1 | 2,250 | 3,000 | 2,800 | 2,800 |
| QRsP-2 | 8,200 | 8,800 | 8,700 | 8,800 |
| QRsP-3 | 8,000 | 7,500 | 7,400 | 7,500 |
| QRsP-4 | 6,600 | 6,800 | 6,800 | 6,800 |

[a]QR-32 cells and QRsP tumour lines were maintained under normal culture conditions (See Materials and methods) for the indicated times. [b]Mice were injected s.c. with 2 × $10^5$ of one or other type of tumour cell. [c]$PGE_2$-production during *in vitro* culture of QR-32 and QRsP tumour cells was measured by the same procedure as described in Table II-i.

significant differences were observed in their growth rates and doubling times in culture.

*Growth of QR-32 cells after coimplantation with gelatin sponge-reactive peritoneal cells*

To investigate the role of host cells reactive to gelatin sponge in the enhancement of *in vivo* s.c. tumorigenicity, we harvested gelatin sponge-reactive peritoneal exudate cells from mice which had been implanted with gelatin sponge. After QR-32 cells (1 × $10^5$) had been added to gelatin sponge-reactive cells (either 1 × $10^5$ or 1 × $10^6$), cell mixtures were injected s.c. into normal syngeneic mice (Table IV). When QR-32 cells were coimplanted with 1 × $10^5$ or 1 × $10^6$ gelatin sponge-reactive cells, the respective QR-32 cells grew progressively in two out of six animals ($P<0.05$) and five out of 12 animals ($P<0.01$). In none of the groups tested were any significant differences observed in the mean survival times (MST) of the mice which died. As a negative control, 1 × $10^5$ QR-32 cells alone were injected subcutaneously into 20 mice: no resultant tumours were observed. These observations suggest that for tumorigenicity of the QR-32 cells to be increased, the same cells must be exposed to host cells re-

**Table II** Tumorigenicity and prostaglandin $E_2$-production by QR-32 cells and QRsP tumour lines

| Cells[a] | Tumorigenicity: Subcutaneous injection (Died mice/mice used) | Intravenous injection[e] (No. of colonies per mouse lung) | $PGE_2$-production[h] (Mean ± s.d., pg $ml^{-1}$) |
|---|---|---|---|
| QR-32 | 0/8 | 0,0,0,0,0,0,0,0,0,0,2 | 1,467 ± 115 |
| QRsP-1 | 2/5[b] | 20,32,34,85,88,77,115,164[f] | 2,750 ± 433[i] |
| QRsP-2 | 5/5[c] | > 150 × 8[f] | 8,667 ± 416[j] |
| QRsP-3 | 3/5[d] | > 150 × 8[f] | 7,600 ± 529[j] |
| QRsP-4 | 5/5[c] | 0,10,27,29,62,69,77[g] | 6,833 ± 208[j] |

[a]One × $10^5$ QR-32 cells were s.c. coimplanted with gelatin sponge into normal mice. The resultant tumours were cultured separately and named QRsP-1 to QRsP-4. [b,c,d]Normal mice were injected s.c. with 2 × $10^5$ cells of either QRsP tumour cells or QR-32 cell. b; not significant, c; $P<0.01$, d; $P<0.05$, versus animals injected s.c. with QR-32 cells. [e]In a separate experiment, mice were i.v. injected with 1 × $10^6$ of each of the four tumour cell types. 19 days later, the mice were sacrificed and the metastatic nodules on the lung surface were counted microscopically. Each value represents the number of colonies per mouse lung. [f,g]f; $P<0.001$, g; $P<0.05$ versus animals injected i.v. with QR-32 cells. [h]$PGE_2$ levels in supernatants obtained from 1 × $10^5$ tumour cell cultures in 24-well plastic plates in 2 ml of medium for 24 h. Determinations were carried out in triplicate and a mean and standard deviation was obtained. [i,j]i; $P<0.01$, j; $P<0.001$ versus $PGE_2$-production by QR-32 cells alone.

**Table IV** Growth of QR-32 cells after coimplantation with gelatin sponge-reactive host cells

| *No. of QR-32 cells* | *No. of gelatin sponge-reactive cells*[a] | *Tumorigenicity*[b] *Died/used* (%) | *MST of dead mice*[c] |
|---|---|---|---|
| $1 \times 10^5$ | $1 \times 10^5$ | 2/6[d] (33) | 54.5 ± 3.5 |
| $1 \times 10^5$ | $1 \times 10^6$ | 5/12[e] (42) | 50.4 ± 12.2 |
| $1 \times 10^5$ | – | 0/20 (0) | – |

[a]Normal C57BL/6 mice were implanted with gelatin sponge into the peritoneal cavity. Five days later, peritoneal exudate cells were collected and seeded into plastic dishes in medium at 37°C for 1 h. Plastic non-adherent cells were then collected and used as gelatin sponge-reactive host cells. [b]$1 \times 10^5$ QR-32 cells were mixed with or without gelatin sponge-reactive host cells or normal PEC and injected subcutaneously into normal mice. [c]MST; mean survival time in days, (Mean ± s.d.). [d,e]d; $P<0.05$, e; $P<0.01$ versus animals injected with QR-32 cells alone.

**Table VI** Inhibition of increase in $PGE_2$-production by QR-32 cells in the presence of radical scavengers

| *Treated*[a] *with* | *$PGE_2$-production (pg ml$^{-1}$) by*[b] *QR-32 + gelatin sponge-reactive cells* | *QR-32 cells* | *Gelatin sponge-reactive cells* |
|---|---|---|---|
| No scavengers | 8,000 | 580 | 1,900 |
| SOD | 9,200 | 300 | 1,600 |
| Catalase | 620 | <250 | 250 |
| Mannitol | <250 | <250 | <250 |
| SOD + Catalase | 380 | <250 | 250 |
| Indomethacin | <250 | <250 | <250 |

[a]Each cell type ($1 \times 10^4$ QR-32 cells and $1 \times 10^6$ gelatin sponge-reactive cells) was plated into wells in a 24-well plastic plate in 2 ml of medium with or without oxygen radical scavengers; SOD (300 U ml$^{-1}$), catalase (20,000 U ml$^{-1}$), mannitol ($5 \times 10^{-2}$M) for 48 h. [b]$PGE_2$-production after 48 h was measured by the same procedure as described in Table II-i.

active to foreign bodies such as gelatin sponge, which coexist at the site of tumour cell implantation, rather than requiring direct contact with the gelatin sponge for anchorage.

*Increased production of prostaglandin $E_2$ by coculture of QR-32 cells with gelatin sponge-reactive cells*

We measured $PGE_2$-production in the supernatants of cocultures of QR-32 cells ($1 \times 10^4$) and gelatin sponge-reactive cells ($1 \times 10^6$). After 24 and 48 h coculture, $PGE_2$-production increased markedly (Table V). Throughout the experiments, the amount of $PGE_2$ produced by QR-32 cells alone ($1 \times 10^4$) was lower than 1,400 pg ml$^{-1}$, while the $PGE_2$ produced by gelatin sponge-reactive cells ($1 \times 10^6$) was lower than 1,300 pg ml$^{-1}$. We have therefore speculated that the large amount of $PGE_2$ was produced mainly by tumour cells. This speculation is further supported by the fact that QRsP tumour lines produce large amounts of $PGE_2$, even when cultured alone in the absence of host reactive cells (Tables II and III).

*Inhibition of the increase in $PGE_2$-production by QR-32 cells in the presence of radical scavengers*

We also examined the effect of radical scavengers on the large amount of $PGE_2$ produced by QR-32 cells cocultured with gelatin sponge-reactive cells (Table VI). Superoxide dismutase (SOD, 300 U ml$^{-1}$) did not inhibit the production of $PGE_2$ whereas catalase (20,000 U ml$^{-1}$), mannitol ($5 \times 10^{-2}$ M) and SOD plus catalase completely inhibited $PGE_2$-production. As a positive control, marked inhibition of $PGE_2$ synthesis was observed after the addition of the prostaglandin synthesis inhibitor, indomethacin ($10^{-6}$ M).

### Discussion

In this study, we demonstrate that gelatin sponge facilitates the growth of weakly tumorigenic QR-32 fibrosarcoma cells in syngeneic mice. Moreover, QRsP tumour lines established from the resultant tumours exhibited more malignant characteristics as compared with parental QR-32 cells. We thus suspected that gelatin sponge might promote the malignant progression of tumour cells. As possible roles of the gelatin sponge in the facilitation of tumour growth, we first considered that gelatin sponge might provide direct anchorage to tumour cells *in vivo*; second, that the sponge induces host reactive cells at the site of tumour implantation and that the reactive cells promote the growth of tumour cells. The former possibility is unlikely, however, because we found that it is not necessary for the gelatin sponge to exist at the site of tumour implantation for tumour progression to occur. In fact, we observed that QR-32 cells were also able to grow in normal mice when they were coimplanted with gelatin sponge-reactive peritoneal exudate cells (PEC). It can be concluded, therefore, that in our experimental model host cells reactive to gelatin sponge are responsible for the facilitation of the *in vivo* growth of weakly tumorigenic QR-32 cells.

The rationale for using gelatin sponge in our experiments includes the following two points: first, the gelatin sponge induces the recruitment and accumulation of sufficient numbers of host cells at tumour implantation sites (Hanto *et al.*, 1982; Akporiaye *et al.*, 1987; Akporiaye & Kudalore, 1989), whilst eliciting relatively low responses in the host (Carter, 1981; Jenkins *et al.*, 1946) and second, gelatin sponge is now widely used in antitumour surgery as a hemostatic material and during chemoembolisation therapy in patients with liver cancer (Shimamura *et al.*, 1988; Sasaki *et al.*, 1987).

We believe that inflammatory processes might involve the conversion of tumour cells into more malignant cells. This hypothesis is partially supported by our *in vitro* experiments in which we observed that the production of prostaglandin $E_2$ ($PGE_2$) by tumour cells was increased after coculture with host reactive cells. With regard to the role of $PGE_2$-production by QR-32 cells, we have previously reported that large amounts of $PGE_2$ inhibit T cell responses to tumour cells and permit their growth as a result of escaping host immunological surveillance (Okada *et al.*, 1990). In the present experiments, we have also observed that large amounts of $PGE_2$-production by QRsP tumour lines correlated well with their *in vivo* tumorigenicity (Table II).

The emergence of progressor QRsP tumour lines might be due to the pre-existence of progressor cells within the QR-32 tumour cell population which become dominant under appropriate micro-environmental conditions, a process which is generally termed *in vivo* selection pressure (Fidler & Kripke, 1977; Morikawa *et al.*, 1988). Although the *in vivo* selection pressure is likely to be one of the mechanisms responsible for the emergence of progressor tumour cells even in our experiments, we also consider that another possibility involving host reactive cell or their products may increase tumour malignancy through the induction of mutations in tumour

**Table V** Increased production of prostaglandin $E_2$ by coculture of QR-32 cells with gelatin sponge-reactive cells

| *No. of tumour cells* | *No. of reactive cells*[a] | *$PGE_2$-production during observation period*[b] 24 | 48 | *(hours)* |
|---|---|---|---|---|
| $1 \times 10^4$ | $1 \times 10^6$ | 13,500 ± 500 | 15,667 ± 557 | |
| $1 \times 10^4$ | – | 1,383 ± 29 | 1,350 ± 50 | |
| – | $1 \times 10^6$ | 1,167 ± 115 | 947 ± 61 | |

[a]Gelatin sponge-reactive cells were obtained by the same procedure as described in Table IV-a. [b]At 24 and 48 h, the coculture supernatants were assayed for $PGE_2$. Determinations were carried out in triplicate and a mean and standard deviation was obtained (pg ml$^{-1}$).

cells by producing oxygen radicals. This consideration was raised by two of our own observations. First, QR-32 cells were obtained from cloned tumour variants (Ishikawa *et al.*, 1987*a*; Okada *et al.*, 1990). Secondly, when we obtained subclones from the cloned QR-32 cells, all 14 of the subclones spontaneously regressed when implanted into normal mice and the levels of $PGE_2$-production by these subclones were low and in the same range as those produced by the parental QR-32 cells (manuscript in preparation). We are now investigating whether host reactive cells are easily able to induce mutations in QR-32 cells as detected by specific minisatellite probes (Pc-1) for the detection of mutations in DNA fingerprinting. Such hypotheses are also supported by other results, obtained for different tumour and reactive cell systems (Yamashima *et al.*, 1986; Loveless & Heppner, 1983; Fulton *et al.*, 1984; 1988).

In the present experiments, active radicals produced by host cells reactive to gelatin sponge may be involved in the malignant conversion of tumour cells. We are presently attempting to confirm the direct production of active radicals by gelatin sponge-reactive cells and the mechanism of involvement of the former in tumour progression.

This work was supported in part by Grant-in Aid for Cancer Research (63010001, 01010002, 02151002, 02151057 and 63-2) from the Japanese Ministry of Health and Welfare. And we thank Dr Mark Micallef and Mr William Jones for their valuable suggestions and kind English revision of this manuscript.

### References

AKPORIAYE, E.T., SAUNDERS, G.C. & KRAEMER, P.M. (1987). A gelatin sponge model for studying tumor growth: quantitation of tumor cells and leukocytes in the CHO tumor. *Experimentia*, **43**, 589–593.

AKPORIAYE, E.T. & KUDALORE, M.K. (1989). Implantation of a gelatin-sponge as a model for effector recruitment. *Cancer Immumol. Immunother.*, **29**, 199–204.

BOONE, C.W. (1975). Malignant hemangioendotheliomas produced by subcutaneous inoculation of Balb/3T3 cells attached to glass beads. *Science*, **188**, 68–70.

BOONE, C.W., TAKEICHI, N., EATON, S.D.A. & PARNJPE, M. (1979). 'Spontaneous' neoplastic transformation *in vitro*: A form of foreign body (smooth surface) tumorigenesis. *Science*, **204**, 177–179.

CARTER, S.J. (1981). *Tutorial Pharmacy*, Pitman Books, p 425.

FIDLER, I.J. & KRIPKE, M.L. (1977). Metastasis results from preexisting variant cells within a malignant tumor. *Science*, **197**, 893–895.

FULTON, A.M., LOVELESS, S.E. & HEPPNER, G.H. (1984). Mutagenic activity of tumor-associated macrophages in *Salmonella typhimurium* strains TA98 and TA100. *Cancer Res.*, **44**, 4308–4311.

FULTON, A., DORCEY, L. & HEPPNER, G. (1988). Host inflammatory cells and generation of tumor cell diversity. *Adv. Exp. Med. Biol.*, **233**, 15–20.

HANTO, D.W., HOPT, U.T., HOFFMAN, R. & SIMMONS, R.L. (1982). Recruitment of unsensitized circulating lymphocytes to sites of allogeneic cellular interactions. *Transplantation*, **33**, 541–546.

ISHIKAWA, M., OKADA, F., HAMADA, J.-I., HOSOKAWA, M. & KOBAYASHI, H. (1987*a*). Changes in the tumorigenic and metastatic properties of tumor cells treated with quercetin or 5-azacytidine. *Int. J. Cancer*, **39**, 338–342.

ISHIKAWA, M., HOSOKAWA, M., OH-HARA, N., NIHO, Y. & KOBAYASHI, H. (1987*b*). Marked granulocytosis in C57BL/6 mice bearing a transplanted BMT-11 fibrosarcoma. *JNCI*, **78**, 567–571.

JENKINS, H.P., JANDA, R., CLARKE, J. & ILL, C. (1946). Clinical and experimental observations on the use of gelatin sponge or form. *Surgery*, **20**, 124–132.

LOVELESS, S.E. & HEPPNER, G.H. (1983). Tumor-associated macrophages of mouse mammary tumors. I. Differential cytotoxicity of macrophages from metastatic and nonmetastatic tumors. *J. Immunol.*, **131**, 2074–2078.

MORIKAWA, K., WALKER, S.M., JESSUP, J.M. & FIDLER, I.S. (1988). In vivo selection of highly metastatic cells from surgical specimens of different primary human colon carcinomas implanted into nude mice. *Cancer Res.*, **48**, 1943–1948.

OKADA, F., HOSOKAWA, M., HASEGAWA, J., ISHIKAWA, M., CHIBA, I., NAKAMURA, Y. & KOBAYASHI, H. (1990). Regression mechanisms of mouse fibrosarcoma cells after in vitro exposure to quercetin: Diminution of tumorigenicity with a corresponding decrease in the production of prostaglandin $E_2$. *Cancer Immunol. Immunother.*, **31**, 358–364.

SASAKI, Y., IMAOKA, S., KASUGAI, H., FUJITA, M., KAWAMOTO, S., ISHIGURO, S., KOJIMA, J., ISHIKAWA, O., OHIGASHI, H., FURUKAWA, H., KOYAMA, H. & IWANAGA, T. (1987). A new approach to chemo-embolization therapy for hepatoma using ethiodized oil, cisplatin, and gelatin sponge. *Cancer*, **60**, 1194–1203.

SHIMAMURA, Y., GUNVEN, P., TAKENAGA, Y., SHIMIZU, H., SHIMA, Y., AKIMOTO, H., ARITA, K., TAKAHASHI, A., KITAYA, T., MATSUYAMA. T. & HASEGAWA, H. (1988). Combined peripheral and central chemo-embolization of liver tumors. *Cancer*, **61**, 238–242.

YAMASHINA, K., MILLER, B.E. & HEPPNER, G. (1986). Macrophage-mediated induction of drug-resistant variants in a mouse mammary tumor cell line. *Cancer Res.*, **46**, 2396–2401.